# Envisioning Low-Dose Tarlatamab

**DOI:** 10.1016/j.jtocrr.2026.101042

**Published:** 2026-06-30

**Authors:** Molly C. Tokaz, Jim Pantelas, Charles J. Nock, Mark J. Ratain, Garth W. Strohbehn

**Affiliations:** a Veterans Affairs Puget Sound Health Care System, Seattle, Washington; b Division of Oncology, Department of Medicine, University of Washington, Seattle, Washington; c National Lung Cancer Roundtable, American Cancer Society, Atlanta, Georgia; d Louis Stokes Cleveland Department of Veterans Affairs Medical Center, Cleveland, Ohio; e Division of Oncology, Department of Medicine, Case Western Reserve University, Cleveland, Ohio; f Section of Hematology/Oncology, Department of Medicine, The University of Chicago, Chicago, Illinois; g Veterans Affairs Center for Clinical Management Research, Ann Arbor, Michigan; h Rogel Cancer Center, Michigan Medicine, Ann Arbor, Michigan; i LTC Charles S. Kettles VA Medical Center, Ann Arbor, Michigan; j Institute for Health Policy and Innovation, University of Michigan, Ann Arbor, Michigan; k Lung Precision Oncology Program, Charles S. Kettles VA Medical Center, Ann Arbor, Michigan; l Division of Hematology/Oncology, Department of Medicine, University of Michigan, Ann Arbor, Michigan; m Center for Global Health Equity, University of Michigan, Ann Arbor, Michigan

The 2024 regulatory approval of tarlatamab, a bispecific T-cell engager,^1^ created optimism for improved survival outcomes for patients with extensive-stage SCLC (ES-SCLC). Its development coincided with the U.S. Food and Drug Administration’s (FDA) introduction of Project Optimus, a coordinated effort guiding industry-wide cancer drug dosing to optimize efficacy and toxicity.^2^^,^^3^ On the surface, tarlatamab’s sponsor seems to have satisfied the FDA’s guidance for pre-approval dose optimization with the DeLLphi-301 trial, a randomized trial in 2L+ ES-SCLC that compared tarlatamab 10 mg with 100 mg and facilitated regulatory approval at the 10 mg dose.^1^^,^^4^ Yet, the publicly available early phase trial evidence—including the FDA’s own clinical pharmacology review—suggests that lower tarlatamab doses might better balance risk and benefit for patients.

Tarlatamab’s phase 1 dose-escalation study, DeLLphi-300, began enrollment in December 2017.^5^ Participants were allocated, following a traditional phase I non-randomized algorithm, to dose levels of less than 3 mg (*n* = 21), 3 mg (*n* = 9), 10 mg (*n* = 16), 30 mg (*n* = 7), and 100 mg (*n* = 13), to identify the maximum tolerated dose (MTD) for the expansion cohort analysis. The MTD was not reached, and the expansion cohort enrolled 28 participants at the 100-mg dose level. Both FDA review and the DeLLphi-300 publication disclosed similar objective response rate (ORR) across the 3 mg, 10 mg, and 100 mg dose levels (44%, 33%, and 20%, respectively) of tarlatamab.^1^^,^^5^ In addition, DeLLphi-301 failed to demonstrate any statistically significant relationships between dose and other clinically relevant efficacy metrics (including duration of response [DOR], progression-free survival [PFS], and overall survival [OS]).^1^^,^^5^ Taken together, these data suggest that the dose-response curve’s plateau includes 3 mg.^5^

Conversely, the FDA review suggested that lower tarlatamab doses, following a 1-mg step-up dose on cycle 1, day 1, were safer.^1^ FDA review disclosed rates of grade more than or equal to 2 cytokine release syndrome (CRS) to be 9%, 21%, and 26% with 3 mg, 10 mg, and 100 mg doses of tarlatamab, respectively. Rates of any*-*grade CRS were 27%, 61%, and 66%, respectively, strongly suggesting a dose-toxicity relationship.^1^ DeLLphi-301 offered similar results, with 51% and 61% of patients experiencing any-grade CRS with the 10 mg and 100 mg doses. Because 10 mg exhibited lower adverse event (AE) risk than 100 mg without a meaningful decrement in efficacy, 10 mg was selected for DeLLphi-304, the open-label phase 3 trial exploring tarlatamab versus chemotherapy as second-line or later therapy for ES-SCLC.^6^ DeLLphi-304 echoed DeLLphi-300’s safety findings, with 56% of tarlatamab 10 mg recipients experiencing any-grade CRS.^6^ Although the incidence of grade more than or equal to 3 treatment-emergent AEs was lower with tarlatamab than chemotherapy (27% versus 62%), treatment-related serious AEs were clinically comparable. Finally, DeLLphi-304 disclosed, for the first time, chronic treatment-related AEs, including dysgeusia, anorexia, fatigue, and constipation, which affect patients’ quality of life in the long term.^2^ Taken together, it is plausible that lower dose tarlatamab would maintain efficacy but reduce the incidence and severity of acute and chronic AEs.

It is worth exploring why, despite a clear-cut toxicity signal but no clear efficacy signal, only the 10 mg and 100 mg doses were selected for study in DeLLphi-301. First, the sponsor cited preliminary pharmacokinetic data suggesting a correlation between the average serum tarlatamab concentration achieved during the first cycle of therapy (C_avg_) and depth of response.^4^ Yet, the sponsor’s recently published account of dose-finding revealed that the minimum C_avg_ required to achieve a response (<0.100 mcg/mL) was easily achieved with a 3-mg dose.^7^ Second, exposures resulting from clearly ineffective doses (i.e., with no observed radiographic responses) bias regression models,^7^ restricting modeling to only clinically active dose levels as inputs to the response curve’s morphology. In the case of tarlatamab, we anticipate that updated dose- and exposure-response curves derived from modeling with doses more than or equal to 3 mg would essentially be flat, supporting the sufficiency of 3 mg. Third, C_avg_ is an untested process measure for excluding potentially effective lower doses from testing. Even among participants who achieved high drug concentrations, non-response was the modal outcome.^7^ Finally, from an ethical standpoint, the decision to compare only 10 mg and 100 mg in DeLLphi-301 was in part driven by the sponsor’s unilateral declaration, without patient consultation or input, that 10 mg and 100 mg were both “low risk” doses, despite the incidence of grade more than or equal to 2 CRS or immune effector cell–associated neurotoxicity syndrome (ICANS) (which require hospitalization and specialized medications, e.g., tocilizumab) being nearly 30% for both.^4^

For a uniformly fatal disease such as ES-SCLC, it is ethically dubious to expose patients to heightened risks of acute high-grade toxicities and chronic low-grade toxicities—both of which rob them of quality of life and valuable time at home—unless there is a clear-cut evidence of benefit.^8^ Thoughtful thoracic oncologists should therefore question whether tarlatamab’s dose may be to blame and, as such, whether dose reduction could maintain tarlatamab’s efficacy while reducing the risk of harm—particularly when patient advocates were not included in dose selection for DeLLphi-301. It should give us pause that a 30% chance of an inflammatory reaction that warrants hospitalization and/or intensive care unit admission has gone unquestioned.

A more rigorous and patient-centered approach to tarlatamab dose optimization in the post-approval setting is needed to realize the goals of modern drug development.^8^ First, doses less than or equal to 3 mg merit further testing because the available data suggest no meaningful efficacy benefit—and potential for harm—in escalating above 3 mg. Second, tarlatamab’s unique pharmacology as a bispecific T-cell engager calls for a tailored approach in dose selection that accounts for patient safety, tolerability, and real-world usability. Patient-reported outcomes and quality of life are especially important for inclusion, given the subjectivity of “tolerability” for acute low-grade CRS and ICANS and chronic CRS and ICANS toxicity (e.g., neurocognitive decline^9^). Third, informed consent in post-approval dose optimization should explicitly acknowledge that higher tarlatamab doses could drive earlier discontinuation, whereas lower doses may reduce toxicity and enable longer treatment durations. Fourth, post-approval dose-optimization studies should be designed for the real world and include older, comorbid, and otherwise underrepresented populations. Finally, as it relates to real-world use, patients themselves should have a say in weighing potential tradeoffs between efficacy, tolerability, and cost in dose selection, as these are value judgments rather than statistical ones.^10^^,^^11^

To this end, we would propose a rational clinical trial design using broad inclusion criteria (e.g., physician discretion) and transparent consent for an informed, representative patient population with a simple two-arm design comparing a lower dose of tarlatamab (3 mg) to the approved dose (10 mg). The primary aim would be efficacy equivalence with a prespecified clinical margin, with a secondary aim of “composite tolerability” as defined in partnership with patients and patient advocates (e.g., patient-reported outcomes, weighted toxicity). In the case of clinically equivalent efficacy, the burden would be on the 10-mg dose to prove enhanced tolerability. Having failed to prove the optimal dose before approval, it is up to payers and those whose incentives are aligned with reducing patient harm to test lower doses in the post-approval setting.

Tarlatamab has the potential to meaningfully improve outcomes for patients with SCLC, and it has deservedly received credit for providing patients with this disease (and the thoracic oncologists who treat them) with much-needed hope. Like any therapy, though, balancing potential benefits and risks is foundational to providing patients with high-quality, prudent care in accordance with the ethical foundations of FDA’s Project Optimus: “No one has a right to subject patients to unnecessary toxicities.”^12^ Tarlatamab’s sponsor deserves credit for conducting DeLLphi-301, and the FDA deserves credit for (correctly) approving the 10 mg rather than the 100 mg dose. Furthermore, tarlatamab’s sponsor is evaluating alternate doses of single-agent tarlatamab in 2L+ ES-SCLC in an open-label, randomized, phase 2 trial with ORR as the primary outcome (DeLLphi-309). However, the sponsor has not publicly disclosed either the doses or dose schedules, so its potential contribution to this conversation on lower-dose tarlatamab remains unclear.^13^ There are no patient-centered outcomes listed in the design.^13^ Overall, the approval journey of tarlatamab highlights the essential role of patient stakeholder involvement early in clinical trial design to allow for more efficient selection of patient-centric dosing.

Lower doses of tarlatamab may offer equivalent efficacy with fewer hospitalizations and less patient burden. As such, the ethical obligation for the field is to test whether lower doses could be substituted for the approved registrational dose. Just because 10 mg is more optimal than 100 mg does not mean that it is the dose that best balances risks and benefits, and practitioners should be cognizant of the possibility that lower tarlatamab doses (3 mg) might maintain the efficacy of 10 mg while exposing patients to less risk. *This* is the optimum to which Project Optimus and dose optimization should aspire. If tarlatamab is to become a global standard of care in SCLC, it is important to ensure that its real-world utilization offers patients the best chance of living the longest, healthiest, and happiest lives possible. Further optimization of this exciting molecule’s dose—identification of its minimum effective dose—may be the avenue for doing just that.

## CRediT Authorship Contribution Statement

**Molly Tokaz:** Conceptualization, Investigation, Roles/Writing - original draft, Writing - review & editing.

**Jim Pantelas:** Writing - review & editing.

**Charles J. Nock:** Writing - review & editing.

**Mark J. Ratain:** Conceptualization, Writing - review & editing.

**Garth W. Strohbehn:** Conceptualization, Investigation, Roles/Writing - original draft, Writing - review & editing.

## Declaration of Generative AI and AI-Assisted Technologies in the Manuscript Preparation Process

During the preparation of this work, the authors used OpenAI to edit the text to comply with word limits and to improve language flow and OpenEvidence as a cross-reference for literature review. After using these tools, the authors reviewed and edited the content as needed and take full responsibility for the content of the published article.

## Disclosure

Drs. Tokaz, Nock, and Strohbehn are employees of the U.S. federal government; the views expressed are their personal views and do not reflect the U.S. federal government policy. Dr. Ratain reports having contracts with Arnold Ventures; receiving consulting fees from Apotex, Argentum Pharmaceuticals, Astellas, Cerona Therapeutics, Conjupro, EQRx, Nestle, Oscotec, Revolution Medicine, Sandoz, Actavis, Alembic, Aurobindo, Cipla, Dr. Reddy’s Laboratories, Mylan, Hetero Labs, Breckenridge Pharmaceutical, Teva, Shilpa, Accord, MSN, Natco, Nurix Therapeutics, Azurity, Formycon, and Glenmark; having a pending patent for low-dose tocilizumab for COVID-19; having royalties related to UGT1A1 genotyping from Mayo Medical Laboratories; and having board leadership for the Optimal Cancer Care Alliance. Dr. Strohbehn reports receiving consulting fees and having stock in VIVIO Health and serving as the director for Optimal Cancer Care Alliance. Dr. Tokaz, Mr. Pantelas, and Dr. Nock declare no conflict of interest.
